# Diversity Manipulation of Psychrophilic Bacterial Consortia for Improved Biological Treatment of Medium-Strength Wastewater at Low Temperature

**DOI:** 10.3389/fmicb.2020.01490

**Published:** 2020-07-24

**Authors:** Floriana Augelletti, Alexandre Jousset, Spiros N. Agathos, Benoit Stenuit

**Affiliations:** ^1^Laboratory of Bioengineering, Earth and Life Institute, Catholic University of Louvain, Louvain-la-Neuve, Belgium; ^2^Ecology and Biodiversity Group, Institute of Environmental Biology, Utrecht University, Utrecht, Netherlands; ^3^Joint Research Unit of Agropolymer Engineering and Emerging Technologies (IATE, UMR 1208), Polytech Montpellier, University of Montpellier, Montpellier, France

**Keywords:** psychrophiles, synthetic consortia, wastewater treatment, phylogenetic diversity, functional diversity, bioaugmentation

## Abstract

Psychrophilic bacteria are valuable biocatalysts to develop robust bioaugmentation formulations for enhanced wastewater treatment at low temperatures or fluctuating temperature conditions. Here, using different biodiversity indices [based on species richness (SR), phylogenetic diversity (PD) and functional diversity (FD)], we studied the effects of microbial diversity of artificial bacterial consortia on the biomass gross yields (measured through OD_600_) and removal efficiency of soluble chemical oxygen demand (mg sCOD removed/mg sCOD introduced) in synthetic, medium-strength wastewater. We built artificial consortia out of one to six bacterial strains isolated at 4°C through combinatorial biodiversity experiments. Increasing species richness resulted in improved sCOD removal efficiency (i.e., 0.266 ± 0.146, 0.542 ± 0.155, 0.742 ± 0.136, 0.822 ± 0.019 for mono-, tri-, penta-and hexacultures, respectively) and higher biomass gross yields (i.e., 0.065 ± 0.052, 0.132 ± 0.046, 0.173 ± 0.049, 0.216 ± 0.019 for mono-, tri-, penta,- and hexacultures, respectively). This positive relationship between biodiversity, sCOD removal and biomass gross yield was also observed when considering metabolic profiling (functional diversity) or evolutionary relationships (phylogenetic diversity). The positive effect of biodiversity on sCOD removal efficiency could be attributed to the selection of a particular, best-performing species (i.e., *Pedobacter* sp.) as well as complementary use of carbon resources among consortia members (i.e., complementarity effects). Among the biodiversity indices, PD diversity metrics explained higher variation in sCOD removal than SR and FD diversity metrics. For a more effective bioaugmentation, our results stress the importance of using phylogenetically diverse consortia, with an increased degradation ability, instead of single pure cultures. Moreover, PD could be used as an assembly rule to guide the composition of mixed cultures for wastewater bioaugmentation under psychrophilic conditions.

## Introduction

Wastewater temperature is a key parameter influencing microbial functions in aerobic wastewater treatment processes (Zhou et al., 2018). Due to climate seasonal changes or differences in geographic area, wastewater temperature can decrease below 10°C, resulting in a reduction of biological activity and reaction rates, as described by the van't Hoff-Arrhenius equation, and in a lower chemical oxygen demand (COD) removal efficiency (Tchobanoglous et al., 2014). Bioaugmentation using psychrophiles has been proposed as a strategy to improve the performance of specific clean-up operations of municipal/domestic wastewater at low temperatures (e.g., carbon removal through oxidation and biomass growth) and compensate for impaired activities of mesophilic bacteria (Zhou et al., 2018).

Psychrophiles are cold-adapted microorganisms living at temperatures close to the freezing point of water, with an optimum growth temperature below 15°C and an upper cardinal temperature of about 20°C (Morita, 1975; Feller and Gerday, 2003). They have the potential to exhibit high metabolic activities at low and moderate temperatures using cold-adaptive traits that compensate for the adverse impact of low temperatures on biochemical reaction rates (Feller and Gerday, 2003; Margesin et al., 2005). The use of psychrophilic strains in bioaugmentation processes was already investigated under cold conditions. For example, a cold-adapted *Arthrobacter psychrolactophilus* was able to grow in a synthetic wastewater at 10°C, inducing a complete clarification of the turbid medium with an efficient hydrolysis of proteins, starch and lipids (Gratia et al., 2009). In another study, psychrophilic bacteria and yeasts fully degraded phenol (a typical aromatic toxic pollutant frequently detected in wastewater) at 10°C under fed-batch cultivation (Margesin et al., 2005). The selection of robust and functionally-active bioaugmentation cultures is of primary importance to optimize the invasion success by mastering the propagule pressure and the establishment of exogenous microorganisms in the invaded ecosystems (El Fantroussi and Agathos, 2005). From product development to final application, bioaugmentation formulations typically progress through five life stages, including (i) capture, (ii) production, (iii) establishment, (iv) function, and (v) downstream impacts (Kaminsky et al., 2019). Bioaugmentation attempts have frequently failed due to poor survival of the inoculated strains. The antagonistic effects of the invaded ecosystem conditions on the active inoculated microbial invaders (i.e., microbiostasis Ho and Ko, 1982) is attributed to both biotic and abiotic factors (van Veen et al., 1997; El Fantroussi and Agathos, 2005). Biotic factors include strain selection criteria based on unique features that confer ecological advantages in the ecosystem, magnitude of propagule pressure, competition between inoculant and indigenous populations or predation by protozoa and bacteriophages. Abiotic factors comprise temperature, pH, substrate availability, or presence of toxic compounds. Both the traits of the invader(s) that allow successful invasion (i.e., invader-centric research Ma et al., 2015; Kinnunen et al., 2016) and the properties of the resident community that determine its susceptibility to invasion (i.e., resident community-centric approach Kinnunen et al., 2016, 2018; Mallon et al., 2018) have recently been investigated.

From the invader-centric perspective, the formulation of microbial inoculants based on eco-physiological attributes and the applications of polycultures instead of single strains in isolation (pure cultures) represent two different strategies to improve bioaugmentation, increasing the survival and functional robustness of the introduced strains.

In particular, the use of consortia can ensure higher removal efficiency of contaminants and more robust processes thanks to their higher functional redundancy, diversity and stability (Stenuit and Agathos, 2015; Giri et al., 2020). Indeed, more diverse communities can gain greater benefits from niche opportunities and assimilate a greater proportion of the available resources (Cardinale, 2011). They also show a greater ability to withstand specific disturbances (resistance) or return to their undisturbed structural and functional baseline after perturbation (engineering resilience; Stenuit and Agathos, 2015).

In this study, the effects of increasing diversity of synthetic, psychrophilic consortia was investigated on the removal of soluble chemical oxygen demand (sCOD) and biomass gross yields in synthetic, medium-strength wastewater at 4°C. The designed synthetic wastewater contained diverse carbon sources available as growth substrates to increase resource heterogeneity for heterotrophic microorganisms (i.e., high niche dimensionality). After isolating six bacterial strains for their ability to remove sCOD from synthetic wastewater at 4°C, we assembled synthetic psychrophilic consortia with increasing richness level, using a combinatorial experiment. The removal of sCOD and the biomass gross yields were analyzed over time and used as functional proxies to assess the coupling of biodiversity and microbial ecosystem functioning. We hypothesized that increased bacterial diversity can enhance sCOD removal efficiency and wastewater treatment through complementarity between species as the underlying mechanism for function optimization at high niche dimensionality. In addition, different biodiversity metrics (i.e., species richness (SR), phylogenetic metrics [Faith's phylogenetic diversity (FPD), mean pairwise distance (MPD)] and functional diversity metrics [dendrogram-based functional diversity (dFD), functional dissimilarity (FDis)] were tested for their relevance to the selection and assembly rules of efficient consortia for bioaugmentation.

## Materials and Methods

### Synthetic Wastewater (SW)

The composition of synthetic wastewater (SW) was formulated according to the Population Equivalent, using the per capita mass constituent discharges measured in Wallonia, Belgium (SPGE, Public Water Management Company, updated on the 15th of June, 2018). This corresponds to a Person Load of 180 L domestic effluent per capita per day with a concentration of 750 mg COD L^−1^ (i.e., 135 g COD per capita per day), 55 mg L^−1^ total nitrogen (i.e., 9.9 g N per capita per day) and 11 mg L^−1^ total phosphorus (i.e., 2 g P per capita per day). The formulation of SW in terms of composition and concentration was designed to simulate the characteristics of a real municipal wastewater (Aiyuk and Verstraete, 2004; O'Flaherty and Gray, 2013). The theoretical composition of SW (pH 7.0) was as follows (per liter): 0.16 g COD starch, 0.026 g COD peptone, 0.149 g COD skim milk, 0.066 g COD yeast extract, 0.263 g COD oleic acid, 0.085 g COD acetic acid, 0.056 g urea, 0.043 g NH_4_Cl, 0.044 g KH_2_PO_4_, 0.01 g MgCl_2_, 0.006 g FeSO_4_.7H_2_O, and 0.001 g CaCl_2_ (Table S1). After autoclaving, 0.3% (v/v) of a trace element solution (SL-6) (ZnSO_4_ × 7 H_2_O 0.10 mg L^−1^, MnCl_2_ × 4 H_2_O 0.03 mg L^−1^, H_3_BO_3_ 0.30 mg L^−1^, CoCl_2_ × 6 H_2_O 0.20 mg L^−1^, CuCl_2_ × 2 H_2_O 0.01 mg L^−1^, NiCl_2_ × 6 H_2_O 0.02 mg L^−1^, Na_2_MoO_4_ × 2 H_2_O 0.03 mg L^−1^) and 0.2% (v/v) of vitamin solution (per 100 mL: 10 mg of pyridoxin-HCl, 2 mg of biotin, 2 mg of folic acid, 5 mg of thiamine-HCl, 5 mg of riboflavin, 5 mg of nicotinic acid, 5 mg of Ca-pantothenate, 5 mg of 4-aminobenzoic acid) were added along with the antifungal inhibitor cycloheximide (final concentration of 50 mg L^−1^). The synthetic wastewater was used either as such (liquid medium) or to formulate solid medium agar plates. In the latter case, 15 g L^−1^ agar was added before autoclaving.

### Bacteria Isolation and Identification

The six psychrophilic strains used in this study were isolated from laboratory cold-room facilities and selected for their ability to grow on SW at 4°C. The isolates were identified using the sequencing of almost full-length 16S rRNA genes. The primer pair used to amplify the 16S rRNA gene included the universal bacterial primer pair 27f (5′-AGAGTTTGATCMTGGCTCAG-3′; Lane, 1991) and 1492r (5′-GGTTACCTTGTTACGACTT-3′; Reysenbach et al., 1992). Herculase II Fusion DNA Polymerase (Agilent Technologies) was used following the manufacturer's instructions. The PCR reactions contained 10 μL 5 × Herculase II reaction buffer, 250 μM each dNTP, 250 nM forward and reverse primer (1.25 μL of 10 μM each primer), 1 μL Herculase II Fusion polymerase, 50–200 ng genomic DNA and water to a final volume of 50 μL.

The PCR thermal cycling scheme was: 95°C for 2 min, 30 cycles (95°C for 20 s, 48°C for 20 s, 72°C for 45 s) and a final extension at 72°C for 3 min. The PCR products of the expected size were excised from the agarose gel (around 1,500 bp) and purified using the QIAquick Gel Extraction Kit (Qiagen). All purified PCR products were sequenced at Macrogen Europe (Amsterdam, The Netherlands). Taxonomic identification was carried out using the Basic Local Alignment Search Tool (nucleotide BLAST using the Nucleotide collection (nt) consisting of GenBank, EMBL, DDBJ, PDB and RefSeq sequences; Altschul et al., 1997). The genus name with the highest maximal identity percentage was retained. The bacterial species belonged to the following taxa: *Rhodococcus* sp., *Pedobacter* sp., *Janthinobacterium* sp., *Brevundimonas* sp., *Pseudomonas* sp., and *Arthrobacter* sp. The 16S sequences were deposited in GenBank under the accession numbers MN722456-MN722461 (Table S2).

Bacterial isolates were kept in glycerol stocks at −80°C and grown on R2A agar plates (per liter, 0.5 g proteose peptone No. 3, 0.5 g dextrose, 0.5 g yeast extract, 0.5 g casamino acids, 0.5 g soluble starch, 0.3 g sodium pyruvate, 0.05 g MgSO_4_ × 7H_2_O, 0.3 g K_2_HPO_4_ and 15 g agar Reasoner and Geldreich, 1985) at 4°C before further experiments.

### Combinatorial Biodiversity Experiment

To evaluate the individual and combined effects of the six different strains (number of factors *k* = 6), bacterial species were assembled with a two-level, ½ fractional factorial design with 32 runs (2^6−1^) (Table S3). The ½ parameter states that only a fraction (50%) of the runs defined by the full factorial design is considered in this experiment (the half fraction in the design is equal to 2^*k*−1^). The presence (+1) or absence (−1) of the strains were used as the levels of the factors and biodegradation activity (COD removal efficiency) and biomass gross yields as the responses. The chosen fractional factorial design resolves all two-factor interactions, with the selection of the negative sign associated with the generating rule. In addition, abiotic controls and hexacultures were included in the design to gain more information without excessively increasing the number of laboratory incubations. Each community was present in triplicate, resulting in the monitoring of 114 ecosystems, including the abiotic controls. A single colony of each bacterial culture was picked and grown in R2A broth (R2B). Cells were harvested at mid-exponential phase, washed (3×) in sterile phosphate buffered saline (PBS) (pH 7.2) and adjusted to a concentration of 8 × 10^8^ cells mL^−1^, using a particle counter (Multisizer 3 Coulter Counter, Beckman Coulter, CA, USA). Bacterial cultures were left for maximum 6 h at room temperature before assembling the communities. Mixed synthetic consortia at a concentration of 8 × 10^8^ cells mL^−1^ were assembled in 1.5 mL Eppendorf tubes, using an equal amount of individual cell suspensions. The initial bacterial concentration of each pure and mixed culture was fixed at 2.0 × 10^7^ total cells mL^−1^. To achieve this, 45 μL from each assemblage were inoculated in 1,755 μL of SW in 2 mL microplates (MASTERBLOCK® 96-Well Deep Well Microplates, Greiner Bio-One). To avoid cross-contamination between the wells during the preparation and the sampling, separated empty wells were used (Figure S1). A gas permeable seal (Breathe-Easy® Gas Permeable Sealing Membrane for Microtiter Plates, Diversified Biotech, Dedham, MA) was then placed upon every microplate to enhance gas transfer, keep sterility and avoid contamination in the plate. This seal was replaced after each sampling. Additionally, three negative controls were present in each plate to verify sterile conditions. The total number of microbial ecosystems was 114. The plates were then incubated at 4°C at 120 rpm for 8 days (192 h). Samples were taken at regular intervals (i.e., every 2 days) to measure the biomass growth. To do so, 150 μL was transferred to microtiter plates and growth was assessed through optical density measurements at 600 nm [OD_600_, (PowerWave HT Microplate Spectrophotometer, BioTek, Winooski, VT, USA)]. At the end of the experiments, 1 mL of culture was filtered and used to quantify sCOD using Spectroquant® test kits (Merck Millipore, Germany).

### Single Bacterial Growth Kinetics

Cell growth kinetics of the six bacterial isolates were examined in a batch reactor configuration using SW as the growth medium. Cultures were incubated at 4°C and agitated at 180 rpm on a rotary shaker. Before starting the kinetic studies, pre-cultures of each individual strain were carried out at 4°C in R2B. Cells were harvested at mid-exponential phase by centrifugation (10,000 rpm for 10 min), washed three times in sterile PBS (pH 7.2) and then re-suspended in PBS to obtain a concentrated cell suspension. The strains were individually inoculated in 50 mL SW to give an initial OD_600_ of 0.025. Three biotic replicates were performed for each culture in 250 mL Erlenmeyer flasks. Samples were taken at regular intervals over a period of 8 days and growth was monitored using OD_600_ measurements, plate counting and particle counter. For plate counting, samples were serially diluted in sterile PBS buffer, and plated on R2A agar. The plates were incubated at 15°C for 3–5 days before counting. For the bacteria enumeration through the particle counter, bacterial samples were transferred into the ISOTONE® II diluent (Beckman Coulter, CA, USA) and quantified with a 20 μm aperture tube. Particle counts for the blank sample, consisting only in ISOTONE® II diluent, were subtracted from all bacterial samples.

### Biodegradation Activity and sCOD Removal Efficiency

The biodegradation of carbonaceous constituents was quantified through measurement of sCOD.

(1) $$\begin{array}{l} \text{sCOD removal efficiency }\left(\text{sCOD-RE}\right)=\\ \;\frac{{\left[sCOD\right]}_{\text{initial}}\;-\;{\left[sCOD\right]}_{\text{final}}}{{\left[sCOD\right]}_{\text{initial}}} \end{array}$$

The samples were filtered using 0.2 μm pore-diameter syringe filters (Minisart®, Sartorius AG, Germany) and analyzed with the Spectroquant® test kits (Merck Millipore, Germany). The ranges of measurement used were 10–150 and 25–1,500 mg O_2_ L^−1^, according to the manufacturer's instructions.

### Computation of Diversity Metrics for Bacterial Assemblages

Faith's phylogenetic diversity (FPD) was computed as the sum of all branch lengths of a phylogenetic tree connecting all species in a local community (Faith, 1992; Vellend et al., 2011). The rooted phylogenetic tree was constructed based on almost full-length 16S rRNA gene sequences, using the Unweighted Pair Group Method with Arithmetic Mean (UPGMA) in Geneious 11.1.4 (https://www.geneious.com). The mean pairwise distance (MPD) was also calculated as the average phylogenetic distance between each pair of species in a community (Webb et al., 2002; Vellend et al., 2011). The FPD and MPD were computed with the picante package in Rstudio (Version 1.0.136; Kembel et al., 2010). Functional diversity metrics based on metabolic profiles were assessed using the Biolog Ecoplate (BIOLOG, Hayward, CA, USA). The Ecoplate contains 31 carbon sources (plus one blank) belonging to different chemical families, repeated three times. Every well of the plate also contains a fluorogenic tetrazolium dye (5 cyano-2,3 ditolyl tetrazolium chloride), which is reduced to a violet-fluorescent formazan molecule when the bacterial cells oxidize the carbon source (Gravel et al., 2011). Briefly, overnight cultures were washed twice in PBS, adjusted to an OD_600_ of 0.05 and added to the plates (150 μL per well). The plates were incubated at 4°C and the color development on each substrate, indicative of bacterial activity, was measured at 590 nm (Jousset et al., 2011). The average OD_590_ values for each substrate were first subtracted from the blank (AU_substrate_). Each AU_substrate_ value was first normalized by the number of electron equivalents (eeq) of the corresponding substrate in each well (AU_substrate_/eeq_substrate_ = AUeeq_substrate_) and then divided by the average value of the whole plate (AUeeq_substrate_ / AUeeq_plate_).

Two methods were used to measure functional diversity (Salles et al., 2012): the first approach was based on distances (Functional Dissimilarity, FDis Heemsbergen et al., 2004; Jousset et al., 2011) and the second based on a dendrogram [dFD (Petchey and Gaston, 2002)]. FDis is the mean functional distance between each pair of species in trait space; dFD corresponds to the sum of branch lengths of a functional dendrogram connecting species of a community together (Petchey and Gaston, 2006). FDis was computed by using a Euclidean distance matrix to sum the distances for all pairs of species in a community. The same distance matrix was used to perform a hierarchical clustering and build the functional dendrogram to compute dFD. The functions “dist()” and “hclust()” in R were used to generate the two metrics. In addition a physiological profile heatmap was drawn with the “pheatmap()” function (Figure S2).

### Data Analysis

Two complementary metrics using the log response ratio were computed to quantify potential effects of biodiversity (i.e., complementarity and selection effects) that governed the diversity-sCOD degradation and diversity-biomass gross yield couplings.

(2) $$\begin{array}{l} {LR}_{\bar{m}}=\text{ln }(\frac{{\text{sCOD-RE}}_{\text{polyculture}}}{{\text{sCOD-RE}}_{\text{moncultures}}}) \end{array}$$

(3) $$\begin{array}{l} {LR}_{\bar{m}}=\text{ln }(\frac{\text{O}{\text{D}}_{600,\text{polyculture}}}{{\text{OD}}_{600,\text{moncultures}}}) \end{array}$$

(4) $$\begin{array}{l} {LR}_{\hat{m}}=\text{ln }(\frac{{\text{sCOD-RE}}_{\text{polyculture}}}{{\text{sCOD-RE}}_{\text{best-performing monoculture}}}) \end{array}$$

(5) $$\begin{array}{l} {LR}_{\hat{m}}=\text{ln }(\frac{{\text{OD}}_{600,\text{polyculture}}}{{\text{OD}}_{600,\text{best-performing monoculture}}}) \end{array}$$

$LR_{\bar{m}}$ compares the value of the response variable (i.e., sCOD removal efficiency or OD_600_) in a polyculture with the mean value of the response variables of the constituent species grown as monocultures (Cardinale et al., 2006). Positive values of $LR_{\bar{m}}\;$indicate the presence of non-transgressive overyielding, i.e., the community performs better than would be expected from the average performance of the single member species. $LR_{\hat{m}}$ compares the value of the response variable in a polyculture with the value of the constituent species showing the highest value as monoculture (Cardinale et al., 2006). Measuring values of $LR_{\hat{m}}>$ 0 corresponds to testing for the presence of transgressive overyielding, i.e., the community outperforms the best-performing monoculture of the component species. All the statistical analyses were performed using GraphPad Prism software (version 8.3.1, San Diego, CA).

Simple linear regressions were used to test for the relationships between community functioning and the different diversity metrics. Data were tested for normality of residuals using the D'Agostino-Pearson test. Differences in response variables, $LR_{\bar{m}}$ and $LR_{\hat{m}}$ between species richness levels were assessed by means of ordinary One-Way ANOVA or Welch's ANOVA and Kruskal-Wallis test when the ANOVA criteria were not met. For $LR_{\bar{m}}\;$and $LR_{\hat{m}}$, *post hoc* multiple comparison tests were carried out to analyze the statistically significant differences of log response ratios for specific pairs of richness levels.

A screening for the main effects (i.e., individual effect of each factor on a dependent variable) of each strain on the two response variables was computed in JMP® software (version 12.2.0). After fitting a multiple linear regression model including the six strains as predictors, the t-ratio, which is defined as the main effect estimate divided by its standard error, is analyzed to test the null hypothesis. In particular, the t-ratio indicated whether a factor (strain) had a significant influence on the response variables and to which extent. For all statistical analyses, a *p* < 0.05 was used to determine statistical significance.

## Results

### Biodiversity Effects and Relationships Between Diversity Metrics and Functioning

Mean differences of biomass growth (OD_600_ values) and sCOD removal efficiency (sCOD-RE) between each species richness level (i.e., mono-, tri-, penta- and hexacultures) were statistically significant [OD_600_: *H* = 40.28, *df* = 3, *p* < 0.0001; sCOD *F*_(3,95)_ = 35.29, *p* < 0.0001 (Figure S3)]. OD_600_ values (i.e., 0.065 ± 0.052, 0.132 ± 0.046, 0.173 ± 0.049, 0.216 ± 0.019 for mono-, tri-, penta-, and hexacultures, respectively) and sCOD removal efficiency (mg sCOD removed per mg sCOD introduced) (i.e., 0.266 ± 0.146, 0.542 ± 0.155, 0.742 ± 0.136, 0.822 ± 0.019 for mono-, tri-, penta-, and hexacultures, respectively) increased with the species richness level of the cultures (Figure S3).

**Figure 1:**
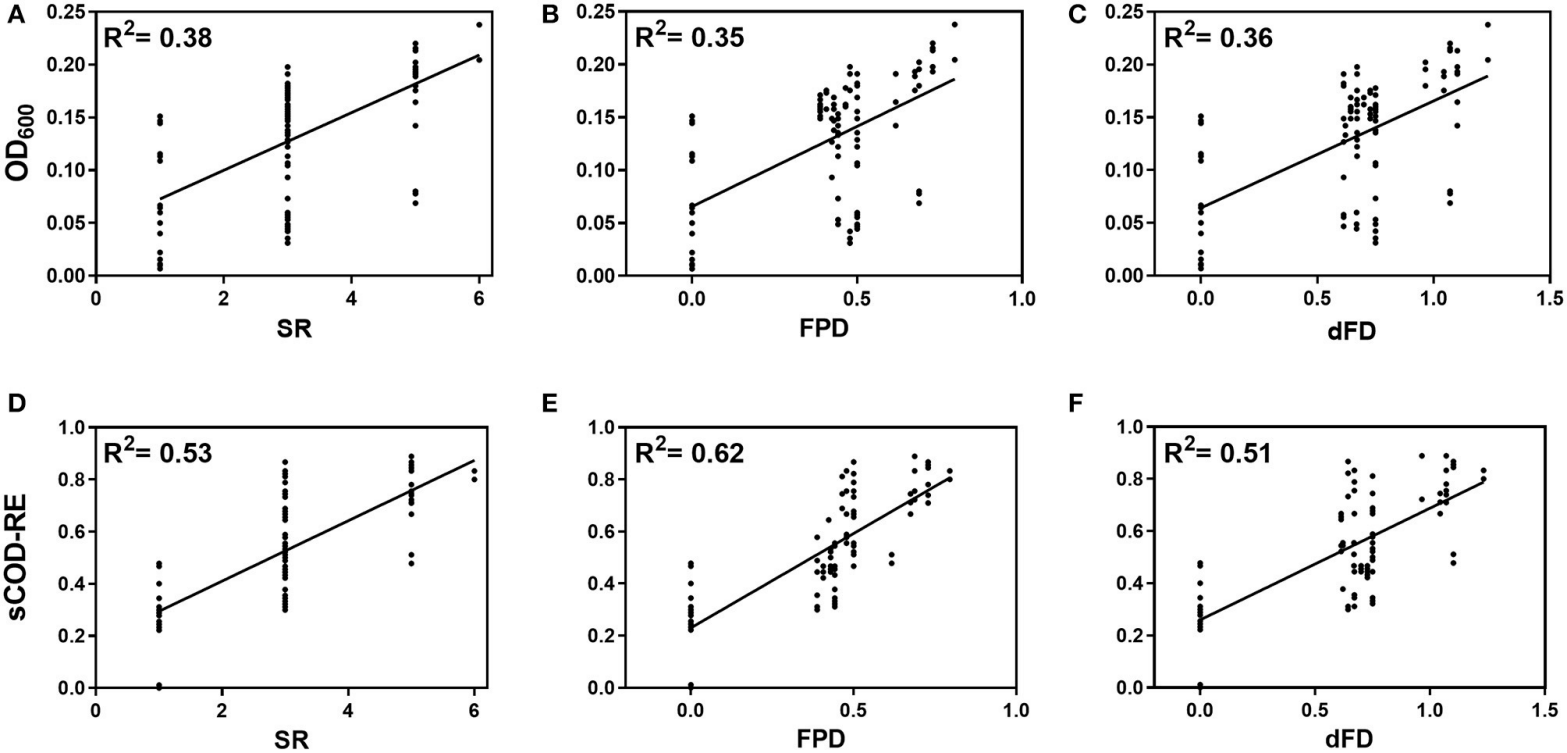
Effect of species richness (SR) **(A, D)**, Faith's phylogenetic diversity (FPD) **(B, E)** and dendrogram-based functional diversity (dFP) **(C, F)** on biomass growth (OD_600_) after a 144-h incubation time **(A–C)** and sCOD removal efficiency (sCOD-RE) at the end of the experiment (192 h) **(D–F)**. The initial OD_600_ in all cultures was adjusted to 2.0 × 10^7^ total cells mL^−1^ and the initial value of sCOD in SW was 540 ± 21 mg sCOD L^−1^. Continuous lines denote simple linear regression significant at *p* < 0.05.

Using simple linear regression, a significant positive relationship between biodiversity dimensions (i.e., SR, FPD, and dFD) and both biomass growth and sCOD removal efficiency was observed [OD_600_: *F*_SR(1,31)_ = 18.75, *p* = 0.0001; *F*_FPD(1,31)_ = 16.44, *p* = 0.0003; *F*_dFD(1,31)_ = 17.80, *p* = 0.0002; sCOD: *F*_SR(1,31)_ = 35.36, *p* < 0.0001; *F*_FPD(1,31)_ = 50.86, *p* < 0.0001; *F*_dFD(1,31)_ = 32.30, *p* < 0.0001; Figures 1A–F]. Using communities with intermediate diversity, i.e., with three and five genotypes only, the increase in functioning with the three indices remained significant only for sCOD removal efficiency [sCOD-RE: *F*_SR(1,24)_ = 8.323, *p* = 0.0081; *F*_FPD(1,24)_ = 22.16, *p* < 0.0001; *F*_dFD(1,24)_ = 5.843, *p* = 0.0236; OD_600_: *F*_SR(1,24)_ = 3.489, *p* = 0.074; *F*_FPD(1,24)_ = 1.812, *p* = 0.1908; *F*_dFD(1,24)_ = 2.583, *p* = 0.1211].

Lower percentages of variance explained by the model were observed for the independent variables SR, FPD and dFD when considering the OD_600_ as the response variable of community functioning (38, 35, and 36%, respectively) compared to sCOD conversion efficiency (53, 62, and 51%, respectively; Figures 1A–F). FPD was the best predictor of sCOD removal with a percentage of variance of 62% explained by the model. Because the models using sCOD conversion efficiency as system function explained higher variances, we focused on this response variable to analyze in more detail the different effects of the independent variables (i.e., diversity metrics).

**Figure 2:**
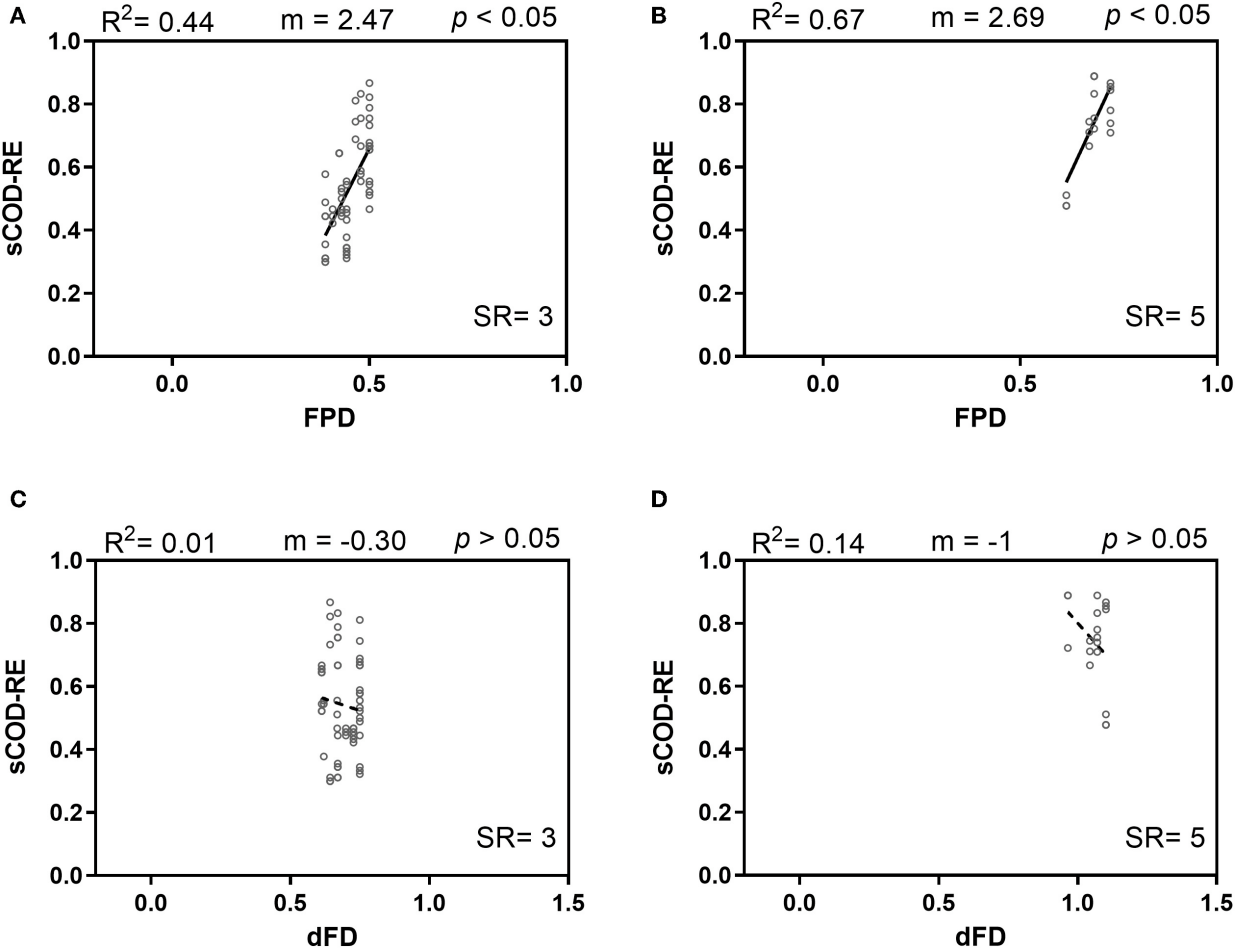
Soluble COD removal efficiency (sCOD-RE) as a function of Faith's phylogenetic diversity (FPD) and dendrogram-based functional diversity (dFD) for intermediate species richness levels: **(A,C)** SR = 3; **(B,D)** SR = 5. Continuous lines indicate simple linear regression significant at *p* < 0.05; dashed lines denote a non-significant simple linear regression (*p* > 0.05).

The strong correlation between SR-FPD and SR-dFD (Figure S4) makes it difficult to properly assess the effect of FPD or dFD *per se* on the sCOD removal efficiency. To disentangle the single effects of FPD and dFD, the data set was first separated into the different SR levels (i.e., *SR* = 3 and *SR* = 5) and the individual effects of FPD and dFD were quantified within the two SR levels. FPD had a significant and positive relationship with sCOD removal for both SR levels [*SR* = 3, *F*_FPD(1,18)_ = 14.30, *p* = 0.0014; *SR* = 5, *F*_FPD(1,4)_ = 7.97, *p* = 0.0477; Figures 2A,B]. Conversely, dFD was negatively related to sCOD removal within both SR levels, although neither of these correlations were statistically significant [*SR* = 3, *F*_dFD(1,18)_ = 0.1931, *p* = 0.6656; *SR* = 5, *F*_dFD(1,4)_ = 0.6473, *p* = 0.4662; Figures 2C,D].

To reduce the collinearity with SR, we also used two other metrics that are less correlated to species richness (Swenson, 2014): mean pairwise distance (MPD, from phylogenetic analysis) and functional dissimilarity (FDis, based on metabolic profile). The lack of correlation between MPD-SR and FDis-SR was also confirmed by directly analyzing the relationships in our experiment through Spearman's correlation (r ~ 0; Figure S4). Significant positive relationship between MPD, FDis and sCOD removal efficiency was observed [*F*_MPD(1,31)_ = 31.49, *p* < 0.0001; *F*_FDis(1,31)_ = 16.76, *p* = 0.0003; Figure S5], with MPD showing a higher explanatory power of the variation in degradation abilities along the biodiversity gradient than FDis (50 and 35%, respectively). Nonetheless, when comparing synthetic consortia with intermediate diversity levels only (*SR* =3 and 5), the increase in sCOD removal remained significant only for MPD [*F*_MPD(1,24)_ = 16.22, *p* = 0.0005; *F*_FDis(1,24)_ = 3.73 × 10^−4^, *p* = 0.9847].

### Mechanisms Underlying the Positive Biodiversity Effects on Microbial Ecosystem Functioning

#### Effect of Species Identities

**Figure 3:**
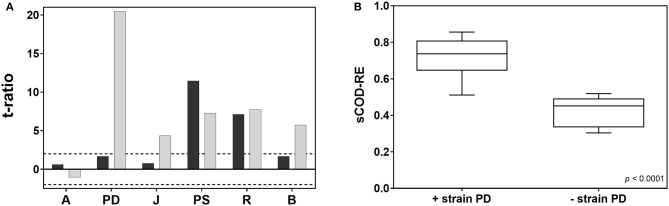
**(A)** Plot of the t-ratio for the estimation of species identity on the cell-density (black bars, 144 h of incubation) and sCOD degradation (gray bars, 192 h of incubation). The horizontal dotted lines indicate the 95% confidence interval. A, *Arthrobacter* sp.; PD, *Pedobacter* sp.; J, *Janthinobacterium* sp.; PS, *Pseudomonas* sp.; R, *Rhodococcus* sp.; B, *Brevundimonas* sp. **(B)** box plot comparing the sCOD degradation activity of synthetic consortia including *Pedobacter* sp. and synthetic consortia without *Pedobacter* sp. (two-sample *t*-test). The box plot shows the minimum and maximum values, with the box presenting the median and the quartiles.

To analyze the effect of species identity on the coupling of biodiversity and functioning, a screening for the main effects (i.e., individual effects of each factor) was realized in JMP®. Each species can contribute differently to the targeted function and this is closely related to the selection effect (i.e., higher probability of including particularly productive species that disproportionately contribute to the targeted function in more diverse communities; Loreau, 1998). The plot of each t-ratio is shown in Figure 3A. A different contribution of species identities was observed depending on which response variable was considered as a measure of system functioning. Using the values of OD_600_ after 144 h of incubation as a target function, the strain *Pseudomonas* sp. showed the highest effect, followed by *Rhodococcus* sp. The other strains (i.e., *Pedobacter* sp., *Brevundimonas* sp., *Arthrobacter* sp., and *Janthinobacterium* sp.) did not significantly contribute to biomass gross yield.

On the contrary, concerning the sCOD removal ability [8 days (192 h) after inoculating the synthetic wastewater], a different effect of species identity was observed: *Pedobacter* sp. had the highest effect on sCOD removal, followed by *Rhodococcus, Pseudomonas*, and *Brevundimonas* sp. (Figure 3A).

The activity of *Arthrobacter* sp., considering both OD_600_ and sCOD removal, did not influence the aggregate community property and the whole behavior of the system.

The importance of *Pedobacter* sp. for sCOD degradation is also reflected by the effect of species composition on degradation activity (Figure 3B): the highest degradation activity was observed in communities that contained *Pedobacter* sp.

To explain the differences in species contribution depending on the chosen response variable, we carried out single bacterial growth kinetics on the synthetic wastewater at 4 °C, measuring the OD_600_ and cell density (cells mL^−1^). In most of the cases, time courses of cell growth measured through plate counting did not correspond to those obtained with OD_600_ measurements (Figure S6). In other words, when a stationary phase is detected based on the OD_600_ curve, an exponential growth phase is observed at the same incubation time for the growth curve based on bacterial cell counting on agar plates [i.e., *Brevundimonas* sp. (Figure S6C), *Pedobacter* sp. (Figure S6E)]. Moreover, to a nearly similar value of OD_600_ for two individual strains, different cell concentrations were measured indicating different conversion factors of one OD_600_ unit into cell concentrations (cells mL^−1^) for each individual strain due to different cell morphologies and dynamics of the total cell volume in a sample (Figure S7). This behavior could be explained by taking into account the time profiles of the mean cell volume (Figure S7), computed from the ratio of the volume of cells per mL and the number of cells per mL obtained from the Coulter counter. The mean cell volume of *Rhodococcus* sp. was almost 10-fold higher than the cell volume of the other strains, whereas the mean cell volume of *Brevundimonas* sp. was the smallest. In the specific case of *Pedobacter* sp. and *Brevundimonas* sp., the quasi constant values of OD_600_ (Figures S6E,C) were due to the decrease in volume starting from the mid-exponential phase and a concomitant increase in cell concentrations. The same trend was observed in general for all bacteria.

#### Analysis of Non-transgressive and Transgressive Overyielding (Complementarity Effect)

**Figure 4:**
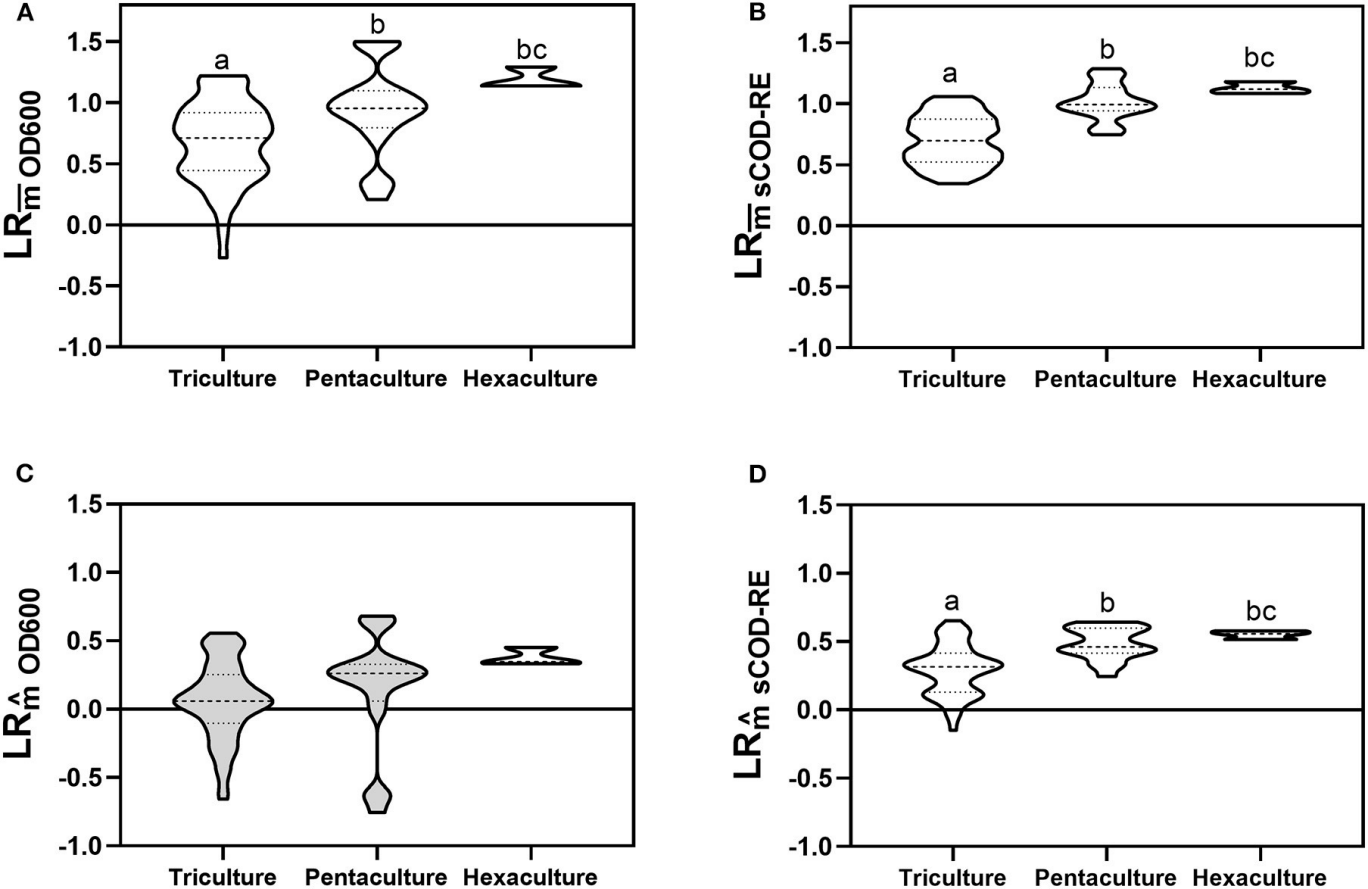
Non-transgressive overyielding values associated with biomass growth **(A)** and sCOD removal efficiency **(B)** for each richness level. Transgressive overyielding values associated with biomass growth **(C)** and sCOD removal **(D)** for each richness level. Values that did not differ significantly from 0 are shown in light gray (one sample *t*-test). Values with different letters indicates statistically significant difference [Tukey's test **(A)**; Dunn's test **(B,C)**; Dunnett's T3 test **(D)**]. Detailed values of non-transgressive and transgressive overyielding for each bacterial consortium are presented in Figure S8. The violin plot shows the minimum and maximum values, with the dashed line representing the median and the dotted lines the quartiles.

We computed both non-transgressive ($LR_{\bar{m}}$) and transgressive overyielding ($LR_{\hat{m}}$) to identify potential complementarity effects that could underlie the observed positive diversity-function couplings (Figures 1A–F). For both biomass growth and sCOD removal activities, we obtained positive values of non-transgressive overyielding for each species richness level (Figures 4A,B). Moreover, there was a significant increase in the mean values between the tri-, penta-, and hexacultures (Tukey's test, Figure 4A; Dunn's test, Figure 4B). However, polycultures can exhibit non-transgressive overyielding through complementarity or selection effect. In order to assess the presence of complementarity effects with more accuracy, we computed the transgressive overyielding. Indeed, polycultures should exhibit transgressive overyielding when both complementarity and selection underpin a positive effect of diversity on ecosystem functioning. Considering the biomass growth, the $LR_{\hat{m}}$ values did not significantly differ from 0 for tri- and pentaculture and the differences between richness levels were not significant (Dunn's test, Figure 4C). Conversely, $LR_{\hat{m}}$ values associated with sCOD removal efficiency were significantly positive for all richness levels and differed significantly comparing the tri-, penta-, and hexacultures (Dunnett's T3 test, Figure 4D).

## Discussion

To study the role of biodiversity on synthetic medium-strength wastewater (SW) treatment under psychrophilic conditions, we constructed artificial microbial communities containing up to six different bacterial isolates. We carried out combinatorial biodiversity experiments using a 2^6−1^ fractional factorial design to correlate microbial ecosystem functioning and biodiversity. The investigated components of biodiversity included species richness and identities, phylogenetic diversity metrics (MPD and FPD indices based on 16S rRNA gene analysis) and functional diversity metrics (FDA and dFD indices obtained from metabolic profiles on Biolog Ecoplate). Artificially constructed ecosystems in sterile laboratory devices were used to study the activities of defined mixed cultures growing on synthetic wastewater under psychrophilic conditions. The response variables comprised sCOD and the optical density at 600 nm (OD_600_) to assess the effects of biodiversity on sCOD conversion efficiency and biomass gross yields. The statistically-significant positive relationships between biodiversity and both sCOD conversion efficiency and biomass gross yields (Figures 1A–F) supported our hypothesis that higher bacterial diversity would result in an improved wastewater treatment, resource utilization efficiency and biomass productivity (Venail and Vives, 2013).

No saturation in functioning was observed with increasing biodiversity (Figures 1A–F), but this trend was probably due to the low diversity and low functional redundancy in the constructed consortia compared to natural ecosystems characterized by a much higher biodiversity difficult to reproduce in laboratory experiments with tractable model communities (Langenheder et al., 2012).

Another limitation of our study is the difficulty to measure individual population density (and so the evolving diversity during the incubation and the final diversity at the end of the experiment in terms of species evenness). Indeed, the relative abundance of populations in the constructed consortia (species evenness) will likely change in the course of incubation. Therefore, our results refer *sensu stricto* to the coupling of initial bacterial diversity (initial richness and evenness) with sCOD conversion efficiency and bacterial growth (Langenheder et al., 2012).

The effects of community diversity indices (SR, FPD, dFD, MPD, and FDis) were analyzed on the two targeted functions. SR, FPD, and dFD explained lower percentages of variance when considering biomass gross yield instead of sCOD removal efficiency. Focusing on the latter function, FPD was the best predictor of community functioning (i.e., it explained the highest percentage of variance (62%) in sCOD removal efficiency). Due to the high correlation between SR and FPD or dFD (Figure S4), we separated the data set into the different SR levels (i.e., *SR* = 3 and 5) to properly investigate the single effect of FPD and dFD on the sCOD removal. Only FPD was positively correlated with degradation ability within individual SR levels (Figure 2). Therefore, this suggests that not only species richness is important in explaining higher system functioning but that, at the same SR level, other diversity metrics, such as FPD, might be more valuable determinants of system functioning. Moreover, a significant variation in sCOD removal efficiency was also explained by phylogenetic diversity measured as MPD, which is less dependent on SR than FPD (Swenson, 2014; Venail et al., 2015) (Figures S4A,C, S5A). Our results supported the rationale that species sharing distant common ancestors are more likely to be more functionally unique, increasing the chance for complementarity and higher overall ecosystem functioning. Previous studies already showed a positive effect of biodiversity driven by phylogenetic diversity and its higher predictive power in explaining ecosystem functioning (Jousset et al., 2011; Venail and Vives, 2013; Galand et al., 2015).

The two indices of functional diversity (dFD and FDis) explained a lower (Figure 1F, Figure S5B) or no variation in sCOD removal (Figures 2C,D). This is probably because the computation of functional diversity metrics is highly dependent on the methodology used to obtain physiological and phenotypic profiles. In this study, we used the Biolog Ecoplates containing 31 different carbon sources to assess the metabolic diversity of individual bacterial isolates. As suggested previously (Wei et al., 2015; Yang et al., 2017), the design of tailor-made resource use profiling plates can be more representative of the ecological niches of the investigated ecosystem by targeting specific relevant resources and phenotypic traits.

Due to the difficulties of measuring individual population density (due to low volume of the system and low biomass), we could not assess the contributions of individual bacterial species to system functioning through the additive partitioning of biodiversity effects (i.e., complementarity and selection effects; Loreau and Hector, 2001). However, we tried to provide a rough quantitative measurement on the relative importance of selection and complementarity effects in our consortia. The analysis of the effect of species identity on the responses allowed us to determine which bacterial species contributed the most to the targeted function and it represented a rough assessment for the presence of a selection effect. Here, the experimental design of combinatorial biodiversity experiments enabled us to study the main effects of each factor used in the fractional factorial design. Species contributed differently to the functionality of the microbial ecosystem depending on which response was considered as a measure of system functioning (Figure 3). Single bacterial growth kinetics on the synthetic wastewater at 4°C revealed that the biomass gross yield response was biased by OD_600_ measurements due to differences in cell volumes between strains. Moreover, the increase in cell concentrations during the kinetics was characterized by a simultaneous decrease in cell volumes that made the measured OD_600_ values less representative of the actual growth. These results demonstrate that OD_600_, often used as a proxy for biomass productivity, might lead to a biased interpretation of data due to morphological differences between bacteria and temporalfluctuations in cell sizes.

Here, we show the importance of carefully choosing the target function to link a specific biodiversity dimension and ecosystem functioning, and the necessity to properly select the variable to measure the community function (e.g., total community biomass). The OD_600_ has often been used as a common method for estimating the concentration of bacterial cells in microbial BEF experiments (Eisenhauer et al., 2013; Awasthi et al., 2014), but the results might be biased due to different cell volume among the different strains or fluctuating cell volumes in the course of time. To analyze the presence of potential complementarity effects that could underpin, along with selection effects, the observed positive diversity-function couplings, we measured the transgressive overyielding ($LR_{\hat{m}}$) for each polyculture. The transgressive overyielding is a widespread measure used in ecology to quantify the co-contribution of both complementarity and selection effect on the increased system functioning. Polycultures can exhibit non-transgressive overyielding through complementarity or selection effect (Schwinning et al., 2014). When both complementarity and selection underlie a positive effect of diversity on ecosystem functioning, polycultures should exhibit transgressive overyielding (Schwinning et al., 2014; Vasseur and Messinger, 2015). The occurrence of transgressive overyielding in all richness levels was observed only for the removal of sCOD (Figure 4D), suggesting that not only selection effect (due to the presence of *Pedobacter* sp., the best-performing strain) but also complementarity effects between species were common in our experiment. This is in accordance with previous studies where the positive relationship between biodiversity and community productivity was driven by the complementarity effect of diversity (Cardinale, 2011; Venail and Vives, 2013).

## Conclusions

In conclusion, microbial ecosystem functioning and robustness can be optimized for biotechnological applications using the rational design of more diverse synthetic consortia, instead of pure cultures. Positive influences of microbial biodiversity on psychrophilic wastewater treatment could be attributed to both complementarity and selection effects. Despite the low diversity levels used in our experiment, phylogenetic diversity turned to be the major determinant of community performance and a valuable predictor of complementary resource utilization between species. Therefore, it could be used as an assembly rule to formulate consortia able to efficiently degrade the wastewater organic load. Further studies should now investigate the use of the best-performing psychrophilic consortium as a bioaugmentation formulation to treat real domestic wastewater in a reactor configuration that simulates continuous-flow wastewater treatment systems.

## Data Availability Statement

The datasets presented in this study can be found in online repositories. The names of the repository/repositories and accession number(s) can be found at: https://www.ncbi.nlm.nih.gov/nuccore/MN722456, https://www.ncbi.nlm.nih.gov/nuccore/MN722457, https://www.ncbi.nlm.nih.gov/nuccore/MN722458, https://www.ncbi.nlm.nih.gov/nuccore/MN722459, https://www.ncbi.nlm.nih.gov/nuccore/MN722460, https://www.ncbi.nlm.nih.gov/nuccore/MN722461.

## Author Contributions

FA conducted the experiments and drafted the manuscript. FA and BS performed the experimental set-up design and planning, data processing and analysis. All authors contributed to the interpretation of data, to the discussion of the results, and to the writing of the manuscript.

## Conflict of Interest

The authors declare that the research was conducted in the absence of any commercial or financial relationships that could be construed as a potential conflict of interest.

## References

[B1] Aiyuk S. Verstraete W. (2004). Sedimentological evolution in an UASB treating SYNTHES, a new representative synthetic sewage, at low loading rates. Bioresour. Technol. 93, 269–278. 10.1016/j.biortech.2003.11.006 15062822

[B2] Altschul S. F. Madden T. L. Schäffer A. A. Zhang J. Zhang Z. Miller W.. (1997). Gapped BLAST and PSI-BLAST: a new generation of protein database search programs. Nucleic Acids Res. 25, 3389–3402. 10.1093/nar/25.17.3389 9254694 PMC146917

[B3] Awasthi A. Singh M. Soni S. K. Singh R. Kalra A. (2014). Biodiversity acts as insurance of productivity of bacterial communities under abiotic perturbations. ISME J. 8, 2445–2452. 10.1038/ismej.2014.91 24926862 PMC4260711

[B4] Cardinale B. J. (2011). Biodiversity improves water quality through niche partitioning. Nature 472, 86–89. 10.1038/nature09904 21475199

[B5] Cardinale B. J. Srivastava D. S. Emmett Duffy J. Wright J. P. Downing A. L. Sankaran M.. (2006). Effects of biodiversity on the functioning of trophic groups and ecosystems. Nature 443, 989–992. 10.1038/nature05202 17066035

[B6] Eisenhauer N. Schulz W. Scheu S. Jousset A. (2013). Niche dimensionality links biodiversity and invasibility of microbial communities. Funct. Ecol. 27, 282–288. 10.1111/j.1365-2435.2012.02060.x

[B7] El Fantroussi S. Agathos S. N. (2005). Is bioaugmentation a feasible strategy for pollutant removal and site remediation? Curr. Opin. Microbiol. 8, 268–275. 10.1016/j.mib.2005.04.011 15939349

[B8] Faith D. P. (1992). Conservation evaluation and phylogenetic diversity. Biol. Conserv. 61, 1–10. 10.1016/0006-3207(92)91201-3

[B9] Feller G. Gerday C. (2003). Psychrophilic enzymes: hot topics in cold adaptation. Nat. Rev. Microbiol. 1, 200–208. 10.1038/nrmicro773 15035024

[B10] Galand P. E. Salter I. Kalenitchenko D. (2015). Ecosystem productivity is associated with bacterial phylogenetic distance in surface marine waters. Mol. Ecol. 24, 5785–5795. 10.1111/mec.13347 26289961

[B11] Giri S. Shitut S. Kost C. (2020). Harnessing ecological and evolutionary principles to guide the design of microbial production consortia. Curr. Opin. Biotechnol. 62, 228–238. 10.1016/j.copbio.2019.12.012 31954367

[B12] Gratia E. Weekers F. Margesin R. D'Amico S. Thonart P. Feller G. (2009). Selection of a cold-adapted bacterium for bioremediation of wastewater at low temperatures. Extremophiles 13, 763–768. 10.1007/s00792-009-0264-0 19578929

[B13] Gravel D. Bell T. Barbera C. Bouvier T. Pommier T. Venail P.. (2011). Experimental niche evolution alters the strength of the diversity-productivity relationship. Nature 469, 89–92. 10.1038/nature09592 21131946

[B14] Heemsbergen D. A. Berg M. P. Loreau M. van Hal J. R. Faber J. H. Verhoef H. A. (2004). Biodiversity effects on soil processes explained by interspecific functional dissimilarity. Science 306, 1019–1020. 10.1126/science.1101865 15528441

[B15] Ho W. C. Ko W. H. (1982). Characteristics of soil microbiostasis. Soil Biol. Biochem. 14, 589–593. 10.1016/0038-0717(82)90092-X

[B16] Jousset A. Schmid B. Scheu S. Eisenhauer N. (2011). Genotypic richness and dissimilarity opposingly affect ecosystem functioning. Ecol. Lett. 14, 537–545. 10.1111/j.1461-0248.2011.01613.x 21435139

[B17] Kaminsky L. M. Trexler R. V. Malik R. J. Hockett K. L. Bell T. H. (2019). The inherent conflicts in developing soil microbial inoculants. Trends Biotechnol. 37, 140–151. 10.1016/j.tibtech.2018.11.011 30587413

[B18] Kembel S. W. Cowan P. D. Helmus M. R. Cornwell W. K. Morlon H. Ackerly D. D.. (2010). Picante: R tools for integrating phylogenies and ecology. Bioinformatics 26, 1463–1464. 10.1093/bioinformatics/btq166 20395285

[B19] Kinnunen M. Dechesne A. Albrechtsen H. J. Smets B. F. (2018). Stochastic processes govern invasion success in microbial communities when the invader is phylogenetically close to resident bacteria. ISME J. 12, 2748–2756. 10.1038/s41396-018-0202-1 30002504 PMC6194134

[B20] Kinnunen M. Dechesne A. Proctor C. Hammes F. Johnson D. Quintela-Baluja M.. (2016). A conceptual framework for invasion in microbial communities. ISME J. 10, 2773–2775. 10.1038/ismej.2016.75 27137125 PMC5148196

[B21] Lane D. J. (1991). 16S/23S rRNA Sequencing, in Nucleic Acid Techniques in Bacterial Systematics, eds Stackebrandt E. Goodfellow M. (New York, NY: John Wiley & Sons, Inc.), 115–175.

[B22] Langenheder S. Bulling M. T. Prosser J. I. Solan M. (2012). Role of functionally dominant species in varying environmental regimes: evidence for the performance-enhancing effect of biodiversity. BMC Ecol. 12:14. 10.1186/1472-6785-12-14 22846071 PMC3480835

[B23] Loreau M. (1998). Biodiversity and ecosystem functioning: a mechanistic model. Proc. Natl. Acad. Sci.U.S.A. 95, 5632–5636. 10.1073/pnas.95.10.5632 9576935 PMC20430

[B24] Loreau M. Hector A. (2001). Partitioning selection and complementarity in biodiversity experiments. Nature 412, 72–76. 10.1038/35083573 11452308

[B25] Ma C. Liu M. Wang H. Chen C. Fan W. Griffiths B. (2015). Resource utilization capability of bacteria predicts their invasion potential in soil. Soil Biol. Biochem. 81, 287–290. 10.1016/j.soilbio.2014.11.025

[B26] Mallon C. A. Le Roux X. van Doorn G. S. Dini-Andreote F. Poly F. Salles J. F. (2018). The impact of failure: unsuccessful bacterial invasions steer the soil microbial community away from the invader's niche. ISME J. 12, 728–741. 10.1038/s41396-017-0003-y 29374268 PMC5864238

[B27] Margesin R. Fonteyne P. A. Redl B. (2005). Low-temperature biodegradation of high amounts of phenol by *Rhodococcus* spp. and basidiomycetous yeasts. Res. Microbiol. 156, 68–75. 10.1016/j.resmic.2004.08.002 15636749

[B28] Morita R. Y. (1975). Psychrophilic bacteria. Bacteriol. Rev. 39, 144–167. 10.1128/MMBR.39.2.144-167.1975 1095004 PMC413900

[B29] O'Flaherty E. Gray N. F. (2013). A comparative analysis of the characteristics of a range of real and synthetic wastewaters. Environ. Sci. Pollut. Res. Int. 20, 8813–8830. 10.1007/s11356-013-1863-y 23740303

[B30] Petchey O. L. Gaston K. J. (2002). Functional diversity (FD), species richness and community composition. Ecol. Lett. 5, 402–411. 10.1046/j.1461-0248.2002.00339.x

[B31] Petchey O. L. Gaston K. J. (2006). Functional diversity: back to basics and looking forward. Ecol. Lett. 9, 741–58. 10.1111/j.1461-0248.2006.00924.x 16706917

[B32] Reasoner D. J. Geldreich E. E. (1985). A new medium for the enumeration and subculture of bacteria from potable water. Appl. Environ. Microb. 49, 1–7. 10.1128/AEM.49.1.1-7.1985 3883894 PMC238333

[B33] Reysenbach A. L. Giver L. J. Wickham G. S. Pace N. R. (1992). Differential amplification of rRNA genes by polymerase chain reaction. Appl. Environ. Microb. 58, 3417–3418. 10.1128/AEM.58.10.3417-3418.1992 1280061 PMC183115

[B34] Salles J. F. Le Roux X. Poly F. (2012). Relating phylogenetic and functional diversity among denitrifiers and quantifying their capacity to predict community functioning. Front. Microbiol. 3:209. 10.3389/fmicb.2012.00209 22701450 PMC3373147

[B35] Schwinning S. Fox A. G. Kelly K. C. (2014). Temporal niches, ecosystem function and climate change, in Temporal Dynamics and Ecological Process, eds Kelly K. C. Bowler M. G. Fox A. G. (Cambridge: Cambridge University Press), 165–188.

[B36] Stenuit B. Agathos S. N. (2015). Deciphering microbial community robustness through synthetic ecology and molecular systems synecology. Curr. Opin. Biotech. 33, 305–317. 10.1016/j.copbio.2015.03.012 25880923

[B37] Swenson N. (2014). Functional and Phylogenetic Ecology in R. New York, NY: Springer-Verlag.

[B38] Tchobanoglous G. Stensel H. D. Tsuchihashi R. Abu-Orf M. Pfrang W. Bowden G. (2014). Wastewater Engineering. Treatment and Resource Recovery. New York, NY: McGraw-Hill Education, Metcalf and Eddy, Inc., AECOM.

[B39] van Veen J. A. van Overbeek L. S. van Elsas J. D. (1997). Fate and activity of microorganisms introduced into soil. Microbiol. Mol. Biol. R. 61, 121–135. 10.1128/.61.2.121-135.1997 9184007 PMC232604

[B40] Vasseur D. Messinger S. (2015). How does evolutionary history alter the relationship between biodiversity and ecosystem function? in Aquatic Functional Biodiversity: An Ecological and Evolutionary Perspective, eds Belgrano A. Woodward G. Jacob U. (New York, NY: AcademicPress), 53–73.

[B41] Vellend M. Cornwell W. Magnuson-Ford K. Mooers A. (2011). Measuring phylogenetic biodiversity, in Biological Diversity: Frontiers in Measurement and Assessment, eds Magurran A. E. McGill B. J. (New York, NY: Oxford University Press), 194–207.

[B42] Venail P. A. Gross K. Oakley T. H. Narwani A. Allan E. Flombaum P. (2015). Species richness, but not phylogenetic diversity, influences community biomass production and temporal stability in a re-examination of 16 grassland biodiversity studies. Funct. Ecol. 29, 615–626. 10.1111/1365-2435.12432

[B43] Venail P. A. Vives M. J. (2013). Positive effects of bacterial diversity on ecosystem functioning driven by complementarity effects in a bioremediation context. PLoS ONE 8:e72561. 10.1371/journal.pone.0072561 24023751 PMC3762786

[B44] Webb C. O. Ackerly D. D. McPeek M. A. Donoghue M. J. (2002). Phylogenies and community ecology. Annu. Rev. Ecol. Syst. 33, 475–505. 10.1146/annurev.ecolsys.33.010802.150448

[B45] Wei Z. Yang T. Friman V. P. Xu Y. Shen Q. Jousset A. (2015). Trophic network architecture of root-associated bacterial communities determines pathogen invasion and plant health. Nat. Commun. 6:8413. 10.1038/ncomms9413 26400552 PMC4598729

[B46] Yang T. Wei Z. Friman V. P. Xu Y. Shen Q. Kowalchuk G. A.. (2017). Resource availability modulates biodiversity-invasion relationships by altering competitive interactions. Environ. Microbiol. 19, 2984–2991. 10.1111/1462-2920.13708 28229529

[B47] Zhou H. Li X. Xu G. Yu H. (2018). Overview of strategies for enhanced treatment of municipal/domestic wastewater at low temperature. Sci. Total. Environ. 643, 225–237. 10.1016/j.scitotenv.2018.06.100 29936164

