# Supplementary material for: Diversity Manipulation of Psychrophilic Bacterial Consortia for Improved Biological Treatment of Medium-Strength Wastewater at Low Temperature
Source: Front Microbiol. 2020 Jul 24;11:1490. doi: 10.3389/fmicb.2020.01490 (PMC7393979; doi:10.3389/fmicb.2020.01490)
Supplement: Supplementary file 1 [file Data_Sheet_1.docx]

**Supplementary information**

**Table S1.** Synthetic wastewater (SW) composition.

| **Compound** | **MW (g mol^-1^)** | **mg L^-1^** | **mg COD L^-1^** | **ThCOD^#^ or Measured COD conversion factors*** |
| --- | --- | --- | --- | --- |
| Starch  (C_6_H_10_O_5_)_x_ | 162_x_ | 135 | 160 | 1.185 |
| Oleic acid (C_18_H_34_O_2_) | 282.47 | 91 | 263 | 2.89 |
| Acetic acid (C_2_H_4_O_2_) | 60.05 | 80 | 85.3 | 1.066 |
| Peptone | - | 20 | 26 | 1.30* |
| Skim milk | - | 130 | 149.5 | 1.15* |
| Yeast extract | - | 64 | 65.9 | 1.03* |
| Urea (CH_4_N_2_O) | 60.06 | 56.5 | - | - |
| NH_4_Cl | 53.49 | 43.1 | - | - |
| MgCl_2_ | 95.21 | 9.5 | - | - |
| KH_2_PO_4_ | 136.09 | 44.0 | - | - |
| FeSO_4_.7H_2_O | 278.01 | 5.8 | - | - |
| CaCl_2_ | 110.98 | 1.1 | - | - |

^#^ Theoretical chemical oxygen demand (i.e., the stoichiometric amount of oxygen required to fully oxidize a specific compound), expressed as g COD g^-1^ substrate, is calculated using the following expression (C_n_H_a_O_b_N_c_S_d_P_e_ taken as the generic substrate and CO_2_, NH_3_, H_2_O, SO_3_ and P_2_O_5_ taken as the reference states):

$$ThCOD \left( \frac{g COD}{g substrate} \right)= \left[ \frac{4n + 1a - 2b - 3c +6d +5e \left( \frac{eeq}{mol substrate} \right)}{4 \left( \frac{eeq}{mol O_{2}} \right)} \right]\times\left[ \frac{{MW}_{O_{2}} \left( \frac{g O_{2}}{mol O_{2}} \right)}{{MW}_{Substrate} \left( \frac{g substrate}{mol substrate} \right)} \right]$$

* The values for mixed, undefined substrates (peptone, skim milk and yeast extract) were obtained from calibration curves computed for each substrate. Standards at different concentrations (mg of substrate L^-1^) of each substrate were prepared. Their respective COD concentrations were determined spectrophotometrically using the Spectroquant® test kits (Merck Millipore, Germany).

**Table S2.** Phylogenetic affiliations, abbreviations and accession numbers of all bacterial strains isolated in this study.

| **Strain** | **Abbreviation** | **GenBank Accession no.** |
| --- | --- | --- |
| *Rhodococcus* sp. | R | MN722456 |
| *Pseudomonas* sp. | PS | MN722457 |
| *Pedobacter* sp. | PD | MN722458 |
| *Janthinobacterium* sp. | J | MN722459 |
| *Brevundimonas* sp. | B | MN722460 |
| *Arthrobacter* sp. | A | MN722461 |

**Table S3.** Composition of synthetic consortia in the combinatorial biodiversity experiment. +1 means the strain is present and -1 means the strain is absent from the mixture. The screening design was generated in JMP using six continuous factors and a list of fractional factorial designs. The chosen fractional factorial design resolves all two-factor interactions, with the selection of the negative sign associated with the generating rule. In addition, microcosms with 0 and 6 strains were included in the design.

| Mixture Number | Species Richness (SR) | *Arthrobacter* sp. (A) | *Pedobacter* sp. (PD) | *Janthinobacterium* sp. (J) | *Pseudomonas* sp. (PS) | *Rhodococcus* sp. (R) | *Brevundimonas* sp. (B) |
| --- | --- | --- | --- | --- | --- | --- | --- |
| 1 | 1 | +1 | -1 | -1 | -1 | -1 | -1 |
| 2 | 1 | -1 | +1 | -1 | -1 | -1 | -1 |
| 3 | 1 | -1 | -1 | +1 | -1 | -1 | -1 |
| 4 | 1 | -1 | -1 | -1 | +1 | -1 | -1 |
| 5 | 1 | -1 | -1 | -1 | -1 | +1 | -1 |
| 6 | 1 | -1 | -1 | -1 | -1 | -1 | +1 |
| 7 | 3 | +1 | +1 | +1 | -1 | -1 | -1 |
| 8 | 3 | +1 | +1 | -1 | +1 | -1 | -1 |
| 9 | 3 | +1 | +1 | -1 | -1 | +1 | -1 |
| 10 | 3 | +1 | +1 | -1 | -1 | -1 | +1 |
| 11 | 3 | +1 | -1 | +1 | +1 | -1 | -1 |
| 12 | 3 | +1 | -1 | +1 | -1 | +1 | -1 |
| 13 | 3 | +1 | -1 | +1 | -1 | -1 | +1 |
| 14 | 3 | +1 | -1 | -1 | +1 | +1 | -1 |
| 15 | 3 | +1 | -1 | -1 | +1 | -1 | +1 |
| 16 | 3 | +1 | -1 | -1 | -1 | +1 | +1 |
| 17 | 3 | -1 | +1 | +1 | +1 | -1 | -1 |
| 18 | 3 | -1 | +1 | +1 | -1 | +1 | -1 |
| 19 | 3 | -1 | +1 | +1 | -1 | -1 | +1 |
| 20 | 3 | -1 | +1 | -1 | +1 | +1 | -1 |
| 21 | 3 | -1 | +1 | -1 | +1 | -1 | +1 |
| 22 | 3 | -1 | +1 | -1 | -1 | +1 | +1 |
| 23 | 3 | -1 | -1 | +1 | +1 | +1 | -1 |
| 24 | 3 | -1 | -1 | +1 | +1 | -1 | +1 |
| 25 | 3 | -1 | -1 | +1 | -1 | +1 | +1 |
| 26 | 3 | -1 | -1 | -1 | +1 | +1 | +1 |
| 27 | 5 | +1 | +1 | +1 | +1 | +1 | -1 |
| 28 | 5 | +1 | +1 | +1 | +1 | -1 | +1 |
| 29 | 5 | +1 | +1 | +1 | -1 | +1 | +1 |
| 30 | 5 | +1 | +1 | -1 | +1 | +1 | +1 |
| 31 | 5 | +1 | -1 | +1 | +1 | +1 | +1 |
| 32 | 5 | -1 | +1 | +1 | +1 | +1 | +1 |
| 33 | 6 | +1 | +1 | +1 | +1 | +1 | +1 |
| 34 | 0 | -1 | -1 | -1 | -1 | -1 | -1 |


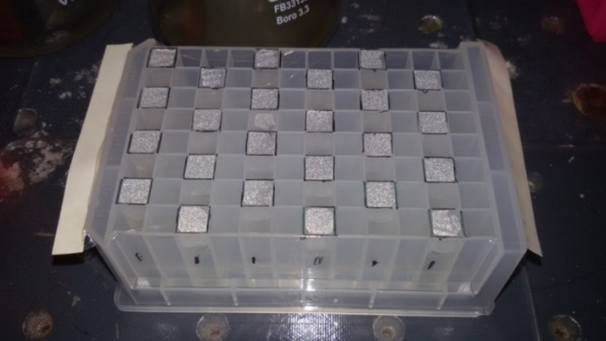


**Figure S1.** Deep-well microplate with a breathable seal. The black squares correspond to the location of the cultures in the plate.


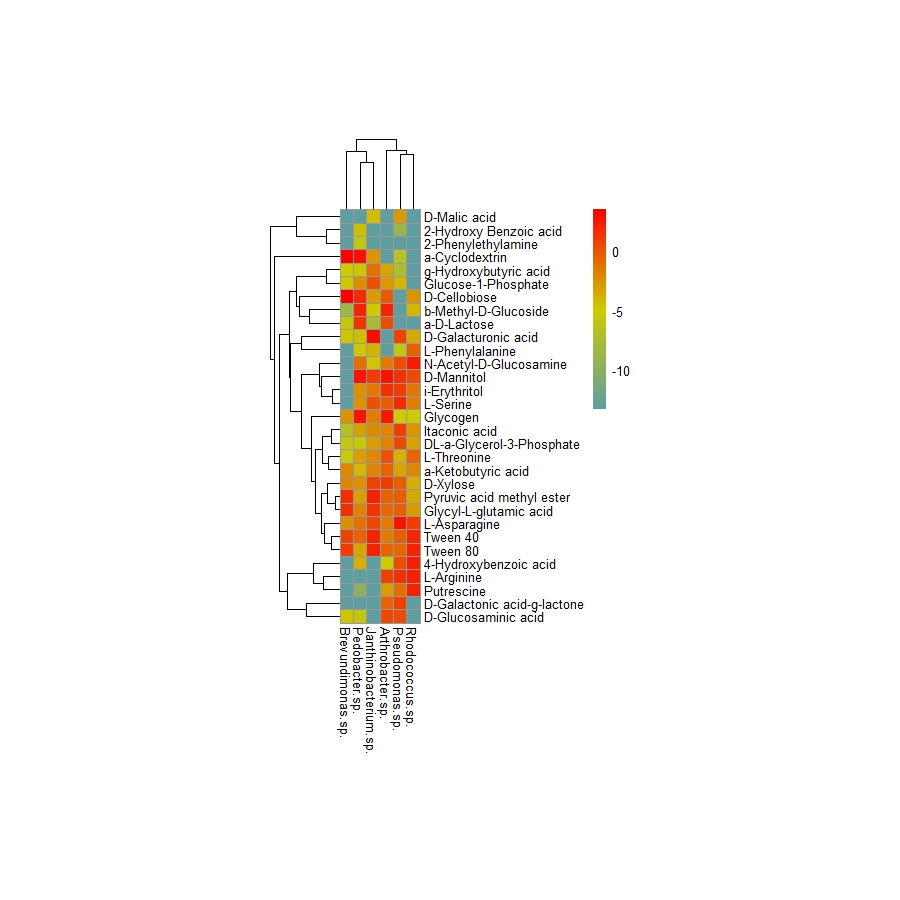


**Figure S2.** Physiological profile heatmap for each strain based on the Biolog Ecoplate assay. Color scale is defined as log_2_ of normalized absorbance values for each substrate.


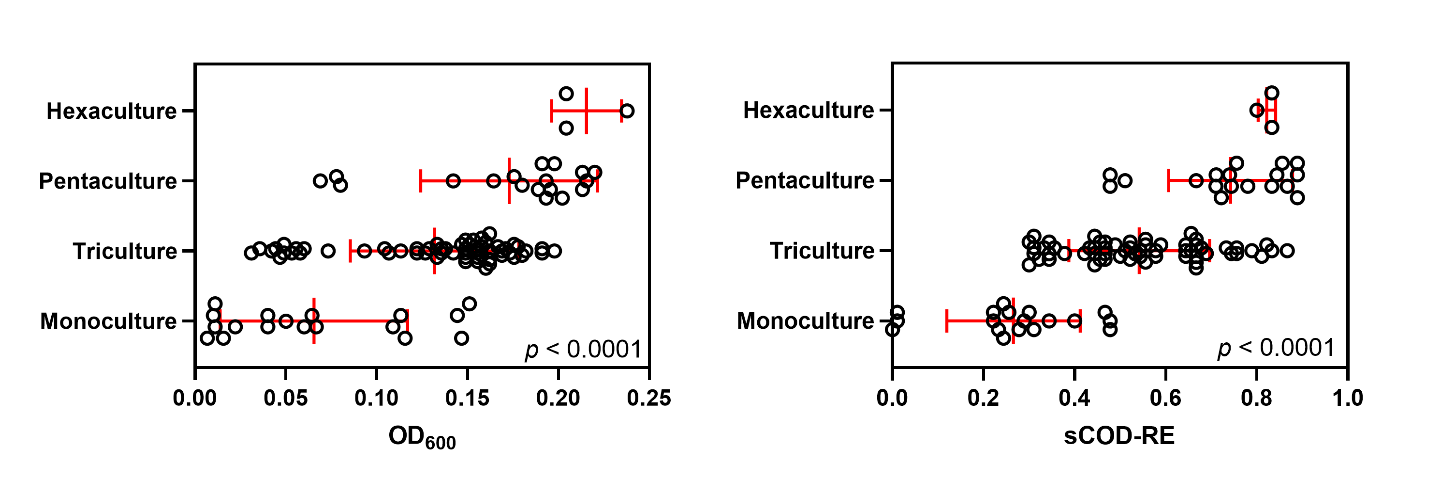


**B**

**A**

**Figure S3.** Mean differences of biomass growth (OD_600_ values, **(A)**) and sCOD removal efficiency (sCOD-RE, **(B)**) between each species richness level. Significant differences between richness levels were assessed by means of Kruskal-Wallis test for biomass growth and ordinary one-way ANOVA for sCOD removal efficiency.

**B**

**A**


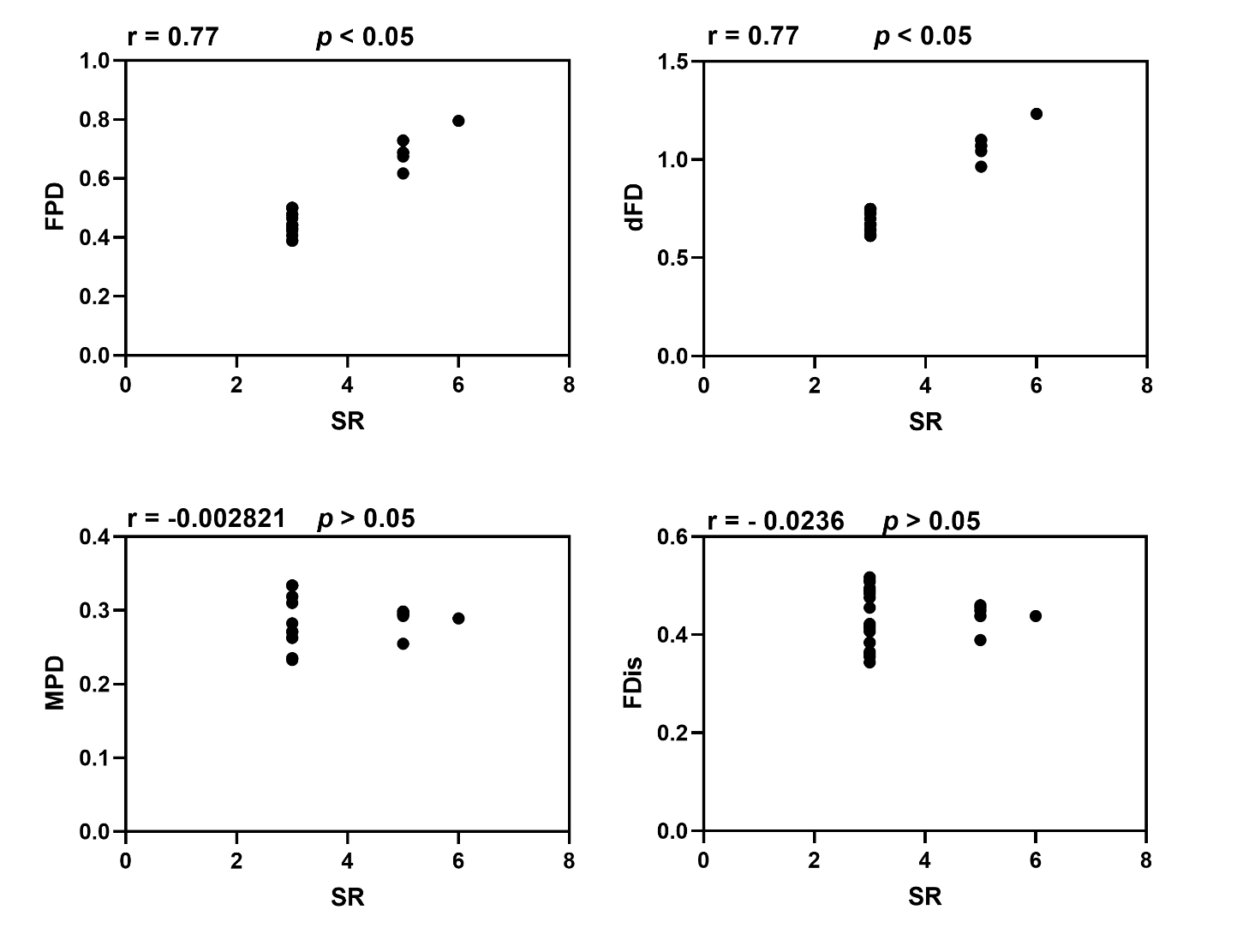


**D**

**C**

**Figure S4.** Correlation between SR and phylogenetic diversity metrics **(A, C)** or functional diversity metrics **(B, D)**. Dendrogram-based diversity metrics (i.e., FPD and dFD) were highly correlated to SR (r > 0.7); distance matrix-based diversity metrics (i.e., MPD and FDis) were independent of SR (r ~ 0). Permutation tests were used to assess if Spearman's correlation coefficients were significantly different from zero.

**B**

**A**

**
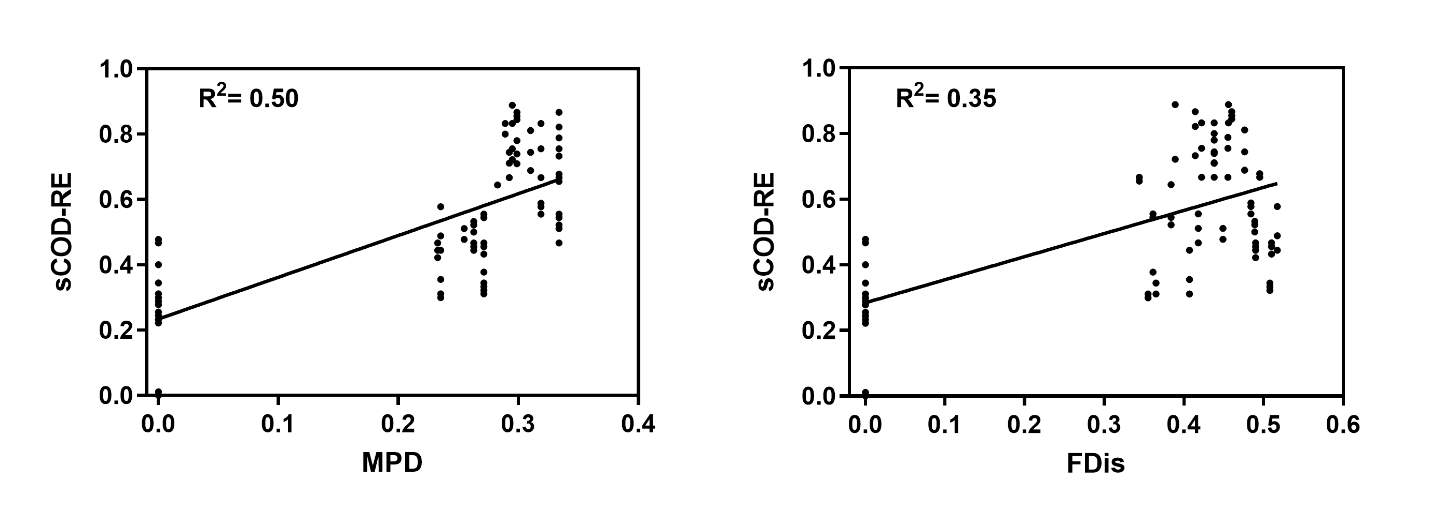
**

**Figure S5.** Effect of mean pairwise distance (MPD) **(A)** and functional dissimilarity (FDis) **(B)** on sCOD removal efficiency. Continuous lines denote simple linear regression significant at *p* < 0.05.

**C**

**A**

**B**

**
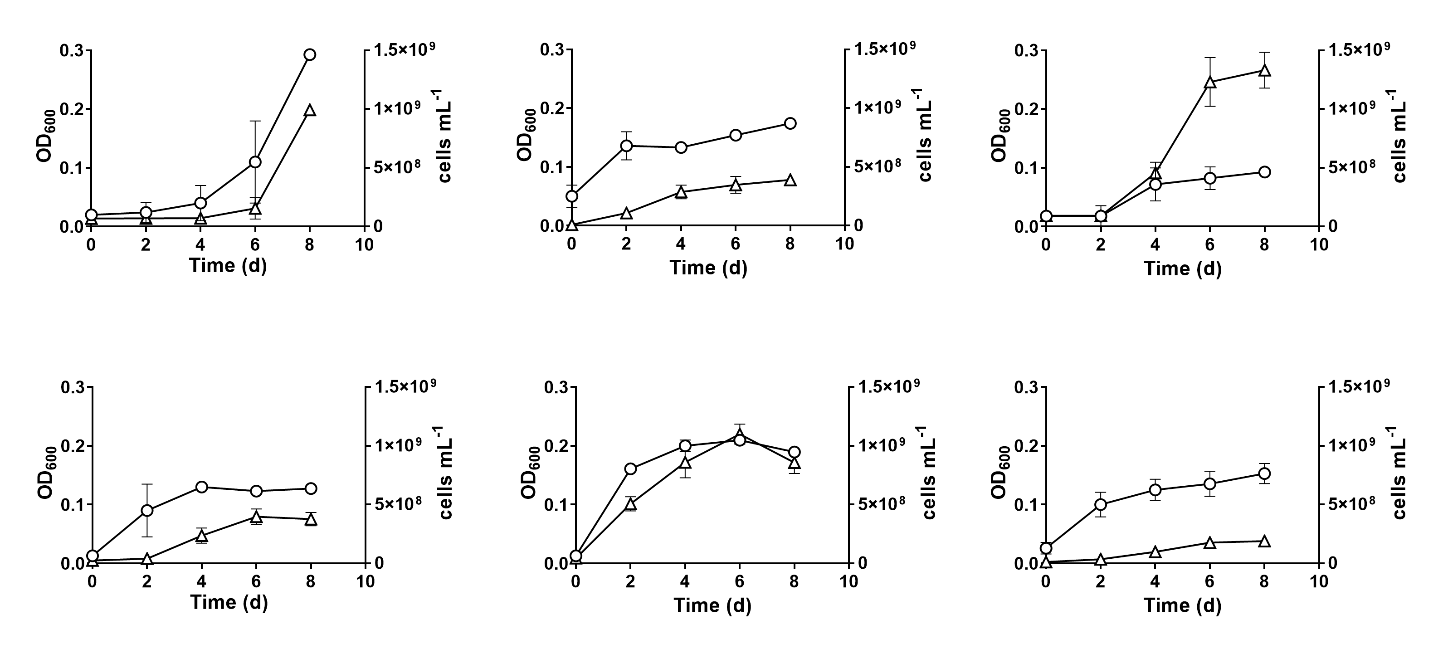
**

**F**

**E**

**D**

**Figure S6.** Time course of bacterial growth (OD_600_ (○) and cell concentration (∆) measured through plate counting) of each strain: **(A)** *Arthrobacter* sp.; **(B)** *Pseudomonas* sp.; **(C)** *Brevundimonas* sp.; **(D)** *Janthinobacterium* sp.; **(E)** *Pedobacter* sp.; **(F)** *Rhodococcus* sp.

**C**

**B**

**A**

*
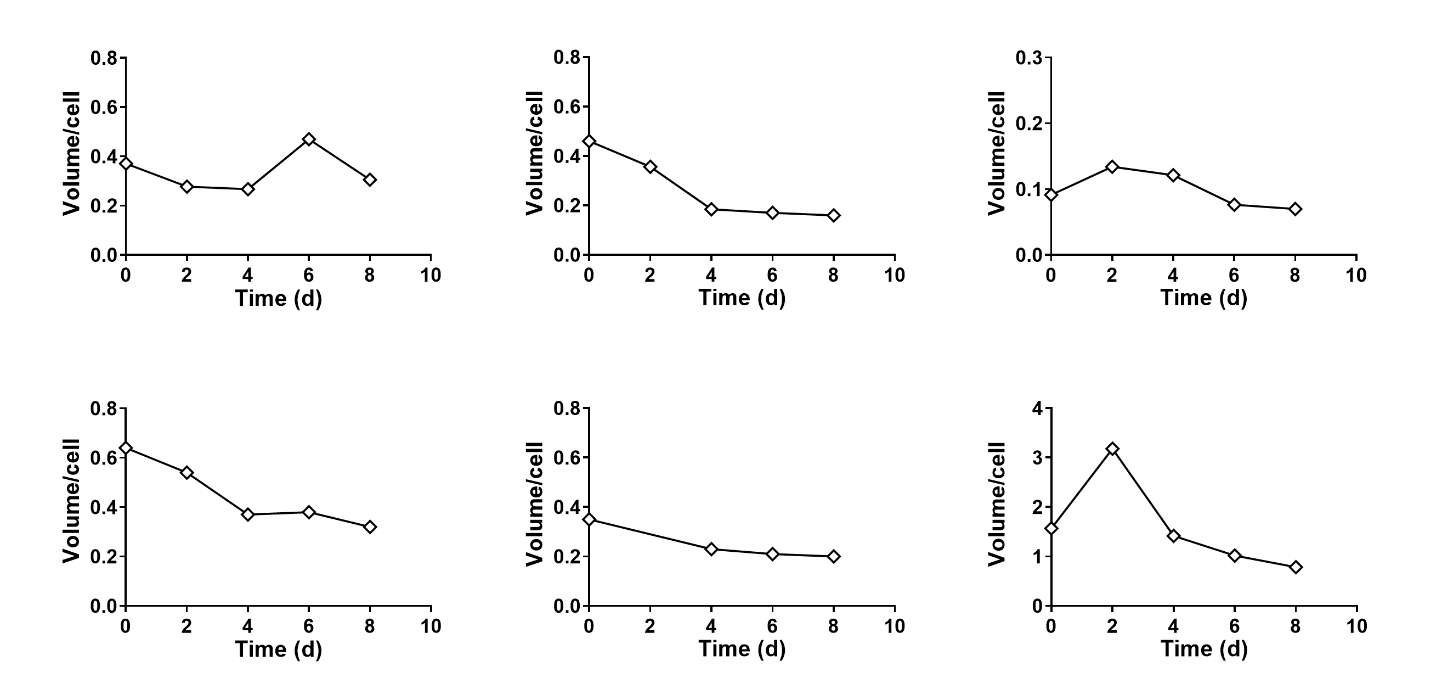
*

**F**

**E**

**D**

**Figure S7.** Time course of averaged cell volume of each strain: **(A)** *Arthrobacter* sp.; **(B)** *Pseudomonas* sp.; **(C)** *Brevundimonas* sp.; **(D)** *Janthinobacterium* sp.; **(E)** *Pedobacter* sp.; **(F)** *Rhodococcus* sp.

**A**

**B**


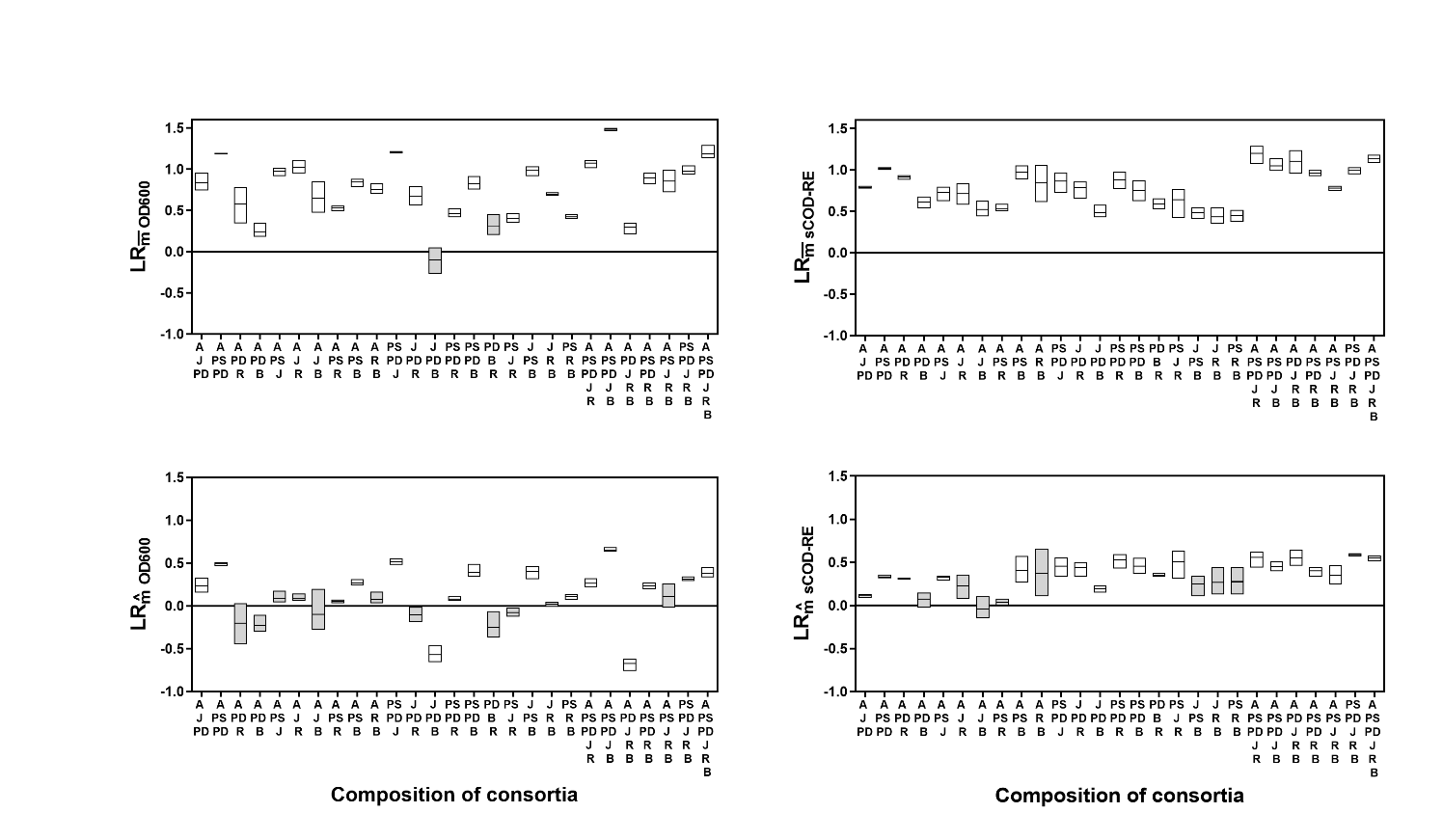


**C**

**D**

**Figure S8.** Non-transgressive overyielding values associated with biomass growth **(A)** and sCOD removal **(B)** for each bacterial consortium. Transgressive overyielding values associated with biomass growth **(C)** and sCOD removal **(D)** for each bacterial consortium. Values that did not differ significantly from 0 are shown in light grey (one sample t-test). *Arthrobacter* sp. (A), *Pedobacter* sp. (PD), *Janthinobacterium* sp. (J), *Pseudomonas* sp. (PS), *Rhodococcus* sp. (R), and *Brevundimonas* sp. (B).
